# Predictors of Psychological Distress in Women with Endometriosis during the COVID-19 Pandemic

**DOI:** 10.3390/ijerph19084927

**Published:** 2022-04-18

**Authors:** Roxana Schwab, Kathrin Stewen, Laura Ost, Tanja Kottmann, Susanne Theis, Tania Elger, Mona Wanda Schmidt, Katharina Anic, Stefanie Roxana Kalb, Walburgis Brenner, Annette Hasenburg

**Affiliations:** 1 Department of Obstetrics and Gynecology, University Medical Center of the Johannes Gutenberg University Mainz, Langenbeckstr. 1, 55131 Mainz, Germany; kathrin.stewen@unimedizin-mainz.de (K.S.); lauost@students.uni-mainz.de (L.O.); susanne.theis2@unimedizin-mainz.de (S.T.); tania.elger@unimedizin-mainz.de (T.E.); mona.schmidt@unimedizin-mainz.de (M.W.S.); katharina.anic@unimedizin-mainz.de (K.A.); stefanie.kalb@unimedizin-mainz.de (S.R.K.); walburgis.brenner@unimedizin-mainz.de (W.B.); annette.hasenburg@unimedizin-mainz.de (A.H.); 2 CRO Dr. Med. Kottmann GmbH & Co. KG, 59077 Hamm, Germany; tk@cro-kottmann.de

**Keywords:** chronic pain, endometriosis, mental health, pain-induced disability, resilience, quarantine, COVID-19

## Abstract

Background: Endometriosis is a multifaceted chronic pain condition that can have a negative impact on mental health. Patients suffering from chronic pain may face an additional psychological burden during adversity, such as the COVID-19 pandemic. The main aim of this research was to evaluate the prevalence of self-reported depression and anxiety, the influence of demographic, endometriosis-specific, pandemic-specific factors, and resilience on mental health outcomes of patients with endometriosis. Methods: An online survey was conducted through patient support groups of women suffering from endometriosis during the first wave of the COVID-19 pandemic. The PHQ-4 questionnaire, which combines two items of the Patient Health Questionnaire for Depression (PHQ-2) and two items from the Generalized Anxiety Disorder Scale (GAD-2) was used to assess self-reported mental health. The Brief Resilience Score (BRS) was employed to evaluate resilience. Independent risk and protective factors for mental health were investigated by multivariate logistic regression analyses. Results: The PHQ-4 questionnaire was completed by 274 respondents. More than 40% reached depression (PHQ-2) and anxiety (GAD-2) scores of ≥3, and more than 20% achieved PHQ-2 and GAD-2 scores of ≥5. High resilience was found to be a reliable and strong independent protector for the probability of developing adverse psychological outcomes: OR 0.295, *p* < 0.001 for developing generalized anxiety disorder (GAD-2 ≥ 3), and OR 0.467, *p* < 0.001 for having major depression (PHQ-2 ≥ 3). Conclusions: Pain-induced disability is an independent risk factor for developing major depression and anxiety, while resilience was identified as a potential protective parameter in terms of positive psychological outcomes in women with endometriosis. The results of this study may help to identify women at risk for adverse mental health outcomes and should encourage healthcare practitioners to establish strategies for the reduction of negative psychological and psychiatric impacts on patients with endometriosis.

## 1. Introduction

Endometriosis is a disease that affects up to 10% of women of fertile age [1]. It is defined by the growth of endometrium-like tissue outside the uterine cavity, which causes a chronic, inflammatory response. Several studies have revealed a diagnostic delay of up to 10.4 years in Germany due to the variety and non-specificity of symptoms [2,3]. As a result, many women experience a variety of symptoms, including chronic pain, pain-induced disability, infertility, and a decline in social and mental well-being [1,2,4,5]. Coexisting conditions and comorbidities frequently result from the chronic nature of the disease and a long delay between the onset of symptoms and diagnosis [1,2]. Aside from an increased risk for certain cancers [1], such as endometrial and endometroid ovarian cancer, and a higher prevalence of autoimmune and atopic conditions [6], the psychological impact on women’s lives is significant [5,7].

Mental health is fundamental to a person’s overall well-being, as it is necessary for personal development and growth [8]. Moreover, physical and mental conditions interact mutually with one another [9]. Women with endometriosis reported more symptoms and were more likely to suffer from depression, anxiety, and emotional distress [4,5,7]. These alterations in mental health were associated with the presence of pain, rather than the diagnosis of endometriosis [10,11,12]. Women with endometriosis were found to have a higher level of depression [4]. Physical pain and other social or contextual vulnerabilities may lead to negative emotional states, and vice versa, as psychological factors, appear to be pain modifiers [4,11,13].

Public health emergencies, such as the coronavirus 2019 pandemic (COVID-19), can significantly impact the physical and psychological well-being of individuals and society [14]. By the end of 2019, the first outbreak of the severe acute respiratory syndrome coronavirus 2 (SARS-CoV-2) was reported in China, and it spread rapidly around the world within a couple of weeks [15,16]. Public health measures such as social distancing, quarantine, and economic lockdown were implemented to prevent the spread of the disease [17], but the above-mentioned preventive measures could on the other side harm mental health [14,17]. Accordingly, women with endometriosis showed an increased social and emotional vulnerability during the COVID-19 pandemic [3], making them more susceptible to mental disorders.

One of the objectives of the present study is to examine the prevalence of self-reported depression and anxiety symptoms among women with endometriosis using the ultra-brief PHQ-4 questionnaire, which can be applied to the daily routine. Moreover, in terms of mental health outcomes, it is crucial for health care professionals to identify and be aware of possible risk factors and potential protectors in women with chronic pain. To our knowledge, this is the first study to assess the impact of sociodemographic aspects, disease-specific variables, pandemic-specific factors, and resilience on self-reported mental health in German women with endometriosis. The findings of this study will aid health care providers and society in reacting more quickly and precisely to prevent adverse mental health outcomes in patients with endometriosis.

## 2. Materials and Methods

### 2.1. Participants of the Study and Recruitment/Sample Population

Between the 6 and 27 of April 2020, an online questionnaire was activated on the Facebook internet platforms of German endometriosis patient support groups. Inclusion criteria were age older than 18 years, diagnosis of endometriosis during a surgical procedure, and informed consent to participate. The questionnaire included questions related to demographic (age, marital status, living alone, and educational level), disease (time since endometriosis diagnosis, age at diagnosis, diagnostic delay of endometriosis, pain characteristics, pain intensity, and pain-induced disability), and pandemic (duration of reduction of the social network, being in isolation or quarantine, and level of reduction of social contacts) parameters.

Pain intensity was assessed via the visual analog pain scale (VAS), a continuous scale ranging from 0 to 100 (100 being the most potent imaginable pain) [18]. Participants were asked to complete a questionnaire with their current pain intensity (VAS_C_) and their pain intensity prior to the implementation of social distancing measures (VAS_P_). For further analyses, pain intensity was evaluated as a continuous variable (VAS_P_, VAS_C_).

Pain-induced disability was explored by the pain disability index (PDI). The reliability of the German version of the PDI questionnaire was described with α = 0.83 [19]. The PDI rates pain-related disability in seven areas of daily life (family/home responsibilities, recreation, social activity, occupation, sexual behavior, self-care, and life-support activity), with each item ranging from 0 (no interference) to 10 (total interference). Basic activities (the sum of self-care and life-support activities) and discretional activities (family/home responsibilities, recreation, social activity, occupation, sexual behavior) were used to create sub-scores. The total score for all items in the global pain-induced disability index can range from 0 to 70 [20]. As mentioned above, participants were asked to answer the questionnaire regarding their current pain-induced disability (PDI_C_) and their pain disability from four weeks prior to the start of social distancing measures (PDI_P_). Pain-induced disability was evaluated as a continuous variable (PDI_P_, PDI_C_).

### 2.2. Patient Health Questionnaire for Depression and Anxiety (PHQ-4)

The Patient Health Questionnaire for Depression and Anxiety (PHQ-4) was employed to assess the psychological burden of the study group. Two items of the Patient Health Questionnaire for Depression (PHQ-2) and the Generalized Anxiety Disorder Scale (GAD-2) were combined to form PHQ-4 [20]. PHQ-4 is an overall screening tool for depression and anxiety. The reliability of the German version of the PHQ-4 was described with α = 0.78 [20]. The assessed by the intercorrelations of the PHQ-4 and its subscales with the Rosenberg Self-Esteem Scale (r = −49 to r = −40), the Questionnaire on Life Satisfaction (r = −39 to r = −32), and the Resilience Scale (r = −35 to r = −28), suggesting construct validity of the measures [20].

Participants used a 4-point Likert scale to respond; ‘not at all’ = 0, ‘several days but less than a week’ = 1, ‘more than half the days’ = 2, and ‘nearly every day’ = 3. PHQ-2, GAD-2, and PHQ-4 scores were computed by adding the scores of individual items [21,22,23,24]. PHQ-2 and GAD-2 scores of ≥3 were considered as cut-off points (“yellow flag”, high probability of depressive or anxiety disorders, as participants were scoring higher than 93% of the normative general population) between the normal range and probable cases of major depression or generalized anxiety. PHQ-2 ≥ 3 has a sensitivity of 82.9% and a specificity of 90.0% for predicting a major depressive disorder [25], and GAD-2 ≥ 3 has a sensitivity of 86.0% and a specificity of 83.0% for predicting a generalized anxiety disorder [26]. PHQ-2 and GAD-2 scores of ≥5 were described as “red flag” (very high probability for depressive or anxiety disorders, as participants were scoring higher than 99% of the normative general population), respectively [24,27].

The overall PHQ-4 score serves as a general marker of psychological distress, indicating symptom burden as well as impairment and disability: a score of 0–2 is considered normal, a score of 3–5 is interpreted as mild, a score of 6–8 is assumed moderate, and a score of 9–12 is related with severe symptoms [24]. Löwe et al. recommended a PHQ-4 score of ≥6 as a “yellow flag” (as participants were scoring higher than 95% of the normative general population) and a PHQ-4 score of ≥9 as a “red flag” (as participants with this score were scoring higher than 99% of the general normative population) for the presence of psychological distress [24,27].

### 2.3. Brief Resilience Scale (BRS)

The BRS was used to assess how resilience affected mental health outcomes. The reliability of the German population was analyzed by Chmitorz and colleagues with α = 0.85, which are in line with the results of the original validation study by Smith and colleagues [28,29]. Smith et al. [28] were the first to describe the BRS, which was developed to identify one’s ability to bounce back from stress. The BRS includes six items that are graded on a 5-point Likert scale: 1 = strong disagreement, 2 = disagreement, 3 = neutrality, 4 = agreement, and 5 = strong agreement. The first, third, and fifth items are positively worded, while the second, fourth, and sixth items are negatively phrased. The average of all six items is applied to calculate the score [29].

### 2.4. Statistical Analysis

The study population was described using descriptive statistics (frequencies, means, and standard deviations) based on their responses to the depression and anxiety screening scale questions. Differences between study respondents and non-respondents were examined by χ^2^-tests and Mann-Whitney-U-tests. The statistical dependence between the rankings of two continuous variables expressed as a correlation coefficient ρ was assessed using Spearman correlations.

Univariate analyses were applied to find variables with proper discriminatory values for binary mental health parameters. The following dependent variables were evaluated: PHQ-2 ≥ 3 (controls (co): PHQ-2 < 3), PHQ-2 ≥ 5 (co: PHQ-2 < 5), GAD-2 ≥ 3 (co: GAD-2 < 3), GAD-2 ≥ 5 (co: GAD-2 < 5), PHQ-4 ≥ 6 (co: PHQ-4 < 6), and PHQ-4 ≥ 9 (co: PHQ-4 < 9). In addition, age ≥ 25 years (co: age < 25 years), having a stable partnership (co: not having a stable partnership), and educational level indicating a tertiary level of education (co: up to a secondary level of education) were used as possible independent demographic predictors. The age cut-off was selected according to previous data that illustrated a high rate of psychological distress in people aged 24 and under [30]. Moreover, pandemic-specific variables were employed as the following: social network large reduction (co: no reduction or mild reduction), period of social distancing ≥ 15 days (co: social distancing period <15 days), isolation or quarantine (co: not being in isolation or quarantine). The cut-off value of 15 days for the social distancing period was chosen in accordance with previously published data on the duration of imposed quarantine [31]. Also, the duration since endometriosis diagnosis (in years, continuous variable), age at diagnosis (in years, continuous variable), duration since pain onset (in years, continuous variable), diagnostic delay (in years, continuous variable), continuous pain (co: patients with pain peaks), and the number of pain localizations (continuous variable, six localizations were assessed) were all applied as potential predictors for disease-specific variables. Pain intensity prior to isolation or quarantine, as well as current pain levels, were included as the following: dysmenorrhea, non-cyclic pain, dyspareunia, dyschezia, dysuria, lower back pain, and general pain (mean pain intensity with regard to all previous named pain localizations).

Variables with *p*-values less than 0.25 in the univariate regression model were then backward stepwise selected into the final multivariate logistic regression model to assess the independence of the above-mentioned predicting variables for predicting mental health outcomes [32,33]. Data were expressed as odds ratio (OR), variance (Nagelkerke R^2^), *p*-value, and 95% confidence interval (95% CI).

All of the tests were two-tailed, with a significance level of *p* < 0.05. All analyses were carried out through SPSS^®^ software Version 24 (IBM Corp. Released 2019. IBM SPSS Statistics for Windows, Version 24.0. Armonk, NY, USA).

## 3. Results

### 3.1. Demographic Characteristics of the Study Group

413 participants met the inclusion criteria and accessed the questionnaire, while 274 (66.34%) attendees answered at least one question about mental health. To better understand the differences between those who answered the questions (group “Respondents”) or those who did not (group “Non-respondents”), the demographic and clinical characteristics of both groups were compared (Table 1). There were no statistically significant differences between the demographic and pandemic-specific variables (Table 1). Respondents showed significantly higher VAS scores for current dysmenorrhea and two items of current pain-induced disability (recreational and professional activities), but there were no significant differences in the current global PDI score between respondents and non-respondents.

### 3.2. Prevalence of Adverse Mental Health Outcomes in Women with Endometriosis and Comparison with German Normative Data

PHQ-2 scores of ≥3 and ≥5 (high and very high probability for depression) were reached by 46.7% (128/274) and 21.5% (59/274) of participants, GAD-2 scores of ≥3 and ≥5 (high and very high probability for anxiety) were achieved by 48.2% (132/274), and 23.7% (65/274) of respondents, respectively. PHQ-4 scores (overall screening for depression and anxiety and total symptom burden) ≥6 and ≥9 were reached by 44.5% (122/274) and 23.4% (64/274) of respondents, respectively. 35.4% (97/274) displayed scores of ≥3 in both sub-scores PHQ-2 and GAD-2 simultaneously, and 13.1% (36/274) showed scores of ≥5 in both sub-scores PHQ-2 and GAD-2.

The mean levels of psychological burden were compared with previously published results in the German population to assess the level of distress in the study population. Based on the findings (Table S1), the study population’s PHQ-2 mean score was 2.83 (SD = 1.69), the mean GAD-2 score was 2.89 (SD = 1.83), and the PHQ-4 average score was 5.72 (SD = 3.21), all of which were significantly higher than previously published studies (all *p*-values < 0.001) [27,34], or subgroups of the German population with chronic self-reported oligo- or multi-localized pain [34]. Moreover, no significant differences were found when comparing the PHQ-2, GAD-2, and PHQ-4 scores of women with endometriosis to fibromyalgia patients [35].

### 3.3. Identification of Predictors of Adverse Mental Health Outcomes

Univariate logistic regression analyses were used to assess the predictability of the selected independent variables on the odds of having a high or very high probability of having a generalized anxiety disorder (GAD-2 ≥ 3, GAD-2 ≥ 5), major depression (PHQ-2 ≥ 3, PHQ-2 ≥ 5), and psychological distress (PHQ-4 ≥ 6, PHQ-4 ≥ 9).

Table 2 shows the impact of demographic variables on self-reported mental health outcomes. A high educational level was found to be a protective factor in all subgroups studied, even if it did not reach statistical significance for those who scored ≥5 points on the PHQ-2 scale. The psychological outcome was unaffected by the participants’ age or status of partnership, or if they were living alone or not.

The influence of pandemic-specific variables on the self-reported outcomes is depicted in Table 3. A significant 2-fold increase in the odds of reporting moderate to high levels of anxiety (GAD-2) and psychological burden (PHQ-4) was observed when the social network was largely reduced.

Table 4, Table 5 and Table 6 show the effect of endometriosis-related medical history, pain intensity, and pain-induced disability on self-reported psychological outcomes. Diagnostic delay, time since pain onset, and duration since endometriosis diagnosis were not significantly linked to negative mental consequences. In univariate analyses, women who reported continuous pain had significantly higher odds of scoring high on the anxiety scale (GAD-2), depression scale (PHQ-2), and overall symptom burden (PHQ-4) (Table 4). Adverse mental outcomes were more strongly associated with current pain intensity than previous pain intensity (Table 5). Current pain-induced global disability, as well as the majority of the current PDI items, were significantly positively correlated with adverse mental outcomes. The probability of a negative mental outcome increased with the severity of the current global disability: the more pronounced the disability, the greater the chance of unfavorable mental effects (Table 6).

High resilience represented to be highly protective against negative mental outcomes, as described by the PHQ-4 scale and the respective sub-scores (Table 7): the women scoring in the highest quartile on the BRS scale (BRS > 3.33) had 92.7% lower odds for the probability of generalized anxiety disorder (GAD ≥ 5), 76.5% lower odds for major depression (PHQ-2 ≥ 5), and 88.4% lower odds for overall very high mental symptom burden (PHQ-4 ≥ 9).

### 3.4. Identification of Independent Predictors of Adverse Mental Health Outcomes

Multivariate logistic regression analysis with the strongest predictors in univariate analyses (*p* < 0.25) of adverse mental outcomes were utilized to assess the independence of the predicting variables (demographic, disease-specific, and pandemic–specific variables, as well as resilience). To avoid model overfitting and information redundancy, the current global pain-induced disability was included in the multivariate analysis, whereas the previous pain-induced disability or previous pain intensity were not involved. Current pain intensity was not considered in the following analyses, because correlation analyses revealed highly significant positive correlations between current global pain-induced disability and current pain intensity, such as current dysmenorrhea (ρ = 0.464; *p* < 0.001), non-cyclic pain (ρ = 0.472; *p* < 0.001), dyspareunia (ρ = 0.354; *p* < 0.001), dysuria (ρ = 0.405; *p* < 0.001), dyschezia (ρ = 0.477; *p* < 0.001), lower back pain (ρ = 0.446; *p* < 0.001), and global pain intensity (ρ = 0.604; *p* < 0.001).

Educational level, duration of isolation or quarantine, reduction of the social network, number of pain localization, global pain-induced disability, and resilience were identified as possible predictors in women with a high probability of experiencing an anxiety disorder, scoring GAD-2 ≥ 3. In the multivariate logistic regression model, isolation or quarantine for more than 14 days (OR 0.295; 95% CI 0.103–0.843; *p* = 0.023) and high resilience (OR 0.241; 95% CI 0.155–0.376; *p* < 0.001) were noticed to protect against high anxiety levels ≥3. In contrast, reduced social network (OR 3.369; 95% CI 1.758–7.760; *p* = 0.001) and high current global pain-induced disability (OR 1.030; 95% CI 1.010–1.051; *p* = 0.003) were risk factors for anxiety subscale scores of 3 or higher. The final regression model (n = 261) explained 37.7% of the variance and demonstrated a sensitivity of 70.5% for predicting GAD-2 ≥ 3 levels.

The multivariate logistic regression model for prediction of a very high probability of general anxiety disorder (GAD-2 ≥ 5) included 261 women and considered the following possible predictors: educational level, endometriosis diagnostic delay, continuous pain, current global pain-induced disability, and resilience. Although current global pain-induced disability emerged as a risk factor for a GAD-2 score of 5 or higher (OR 1.024; 95% CI 1.003–1.045; *p* = 0.027), high resilience was found to be a strong protective factor (OR 0.222; 95% CI 0.133–0.370; *p* < 0.001). The proposed statistical model illustrated 30.0% of the variance. The model had a sensitivity of 78.5% for predicting GAD-2 ≥ 5.

The predictive independence of age, living alone, educational level, reduction of the social network, age at diagnosis of endometriosis, the number of pain localizations, current global pain-induced disability, and resilience were figured out in a multivariate logistic regression model in women with a high probability of major depression, who scored ≥ 3 on the PHQ-2 scale. Protective factors were being 25 years or older (OR 0.461; 95% CI 0.216–0.981; *p* = 0.044) or being resilient (OR 0.467; 95% CI 0.320–0.681; *p* < 0.001). High current global pain-induced disability was a risk factor (OR 1.068; 95% CI 1.046–1.090; *p* < 0.001) for depression subscale scores of 3 or higher. The final model (n = 259) explained 35.2% of the variance in PHQ-2 ≥ 3 scorings and demonstrated a sensitivity of 71.4% in predicting PHQ-2 ≥ 3.

Age, educational level, continuous pain, the number of pain localizations, current global pain-induced disability, and resilience were determined as possible independent variables in women with a very high probability of experiencing major depression (PHQ-2 ≥ 5) and were then entered into the multivariate regression model. Current global pain-induced disability was detected as a risk factor for the depression subscale score of ≥5 (OR 1.048; 95% CI 1.025–1.073; *p* < 0.001), whereas resilience was found to be a potent protective factor (OR 0.340; 95% CI 0.211–0.549; *p* < 0.001). The final multivariable logistic regression model included 261 participants and explained 27.6% of the variance in PHQ-2 ≥ 5 scorings. The sensitivity of PHQ-2 ≥ 5 prediction was 78.5%.

259 participants were involved in the multivariate logistic regression model for the prediction of a high probability of either anxiety or major depression (PHQ-4 ≥ 6). The predictors chosen included age, living alone, educational level, reduction of the social network, age at endometriosis diagnosis, continuous pain, the number of pain localizations, current global pain-induced disability, and resilience. Large reductions in social network (OR 2.654; 95% CI 1.333–5.285; *p* = 0.005) and current global pain-induced disability (OR 1.055; 95% CI 1.034–1.078; *p* < 0.001) were risk factors for high psychological symptom burden as defined by PHQ-4 ≥ 6. Protective parameters for high psychological burden were higher age at diagnosis of endometriosis (OR 0.949; 95% CI 0.904–0.996; *p* = 0.035) and resilience (OR 0.275; 95% CI 0.178–0.424; *p* < 0.001). The final regression model explained 41.2% of the variance in PHQ-4 ≥ 6 scorings and had a prediction sensitivity of 72.2%.

In women with a very high probability of anxiety or major depression, as described by the global score PHQ-4 ≥ 9, the variables educational level, diagnostic delay, continuous pain, the number of pain localizations, current global pain-induced disability, and resilience were analyzed in a multivariate logistic regression model. Current global pain-induced disability proved to be a risk factor for a PHQ-4 scale score of ≥9 (OR 1.053; 95% CI 1.029–1.078; *p* < 0.001). Resilience was detected to be a protective factor (OR 0.286; 95% CI 0.175–0.468; *p* < 0.001). The final multivariable logistic regression model (n = 260) explained 33.3% of the PHQ-4 ≥ 9 scores variance and demonstrated an 80.8% sensitivity for prediction.

## 4. Discussion

According to our results, patients with endometriosis were at a high risk of developing mental symptoms, as 46.7% and 48.2% scored ≥ 3 on the PHQ-2 and GAD-2 scales, indicating a high probability of the presence of major depression and generalized anxiety disorder, respectively. However, a recent study on the average German population performed before the pandemic declared that the prevalence of possible depressive and anxiety disorders was 11.2% and 12.0%, respectively [36]. This finding is in line with that of Löwe et al., who reported a 5.6% overall prevalence for both conditions in the “normal population” using the same self–reported questionnaire PHQ-4, and diagnostic thresholds [27]. Nevertheless, our results are consistent with previous research findings, describing a high prevalence and symptom burden of mental health disorders (up to over 80% prevalence of depressive and anxiety symptoms) in women with chronic disease [5,11,12,36,37,38,39,40,41]. Moreover, the co-occurrence of depression and anxiety in our study collective, defined by both scores ≥3 in 35.4% or both scores ≥5 in 13.1%, was in line with previous findings, indicating co-occurrence in up to 50% of the cases [37]. Even though first-line treatment for depression and anxiety is similar, remission rates in patients who scored high on both conditions may be lower. Thus, it is crucial to identify these patients, as they may benefit from early referral to specialized psychological treatment, closer monitoring, and follow-up [24]. A high level of symptom burden was revealed by 44.5% and 23.4% of women with endometriosis, as measured by PHQ-4 ≥ 6 and PHQ-4 ≥ 9, respectively. High PHQ-4 scores were strongly linked to declines in a variety of functional impairments, such as mental health, social functioning, general health perceptions, role, and physical functioning, bodily pain, mean disability days, and increased healthcare utilization [24]. Our results support the findings of recent studies that women with endometriosis have a higher depressive symptom burden than women without chronic pain [4,10,40], possibly due to the chronic and diverse physical symptoms, paired with the perceived disability and life disadvantages that the affected women face.

A thorough understanding of the risk factors for developing mental health disorders is essential for identifying individuals at risk for depression or anxiety. In univariate analyses, having a lower education (up to secondary level) was considered a risk factor for anxiety (measured by GAD-2) and depression (measured by PHQ-2), as well as for high symptom burden, assessed by the combined score PHQ-4. This data backs up previous findings that reported that a low level of education is a risk factor for the development of mental disorders and that a higher education level can enhance people’s skills and empower more effective coping mechanisms, leading to more favorable psychological outcomes [36,42,43]. These findings are significant because, unlike other factors such as pain intensity, educational level is relatively stable throughout ones’ lifetime. Nevertheless, multivariate analyses revealed that a high academic level was a mediator of mental health rather than an independent protective factor, after adjusting for multiple possible influencing factors. In contrast, age 25 or older proved to be an independent protective factor in women with endometriosis who scored ≥ 3 on the depression subscale PHQ–2, possibly due to more advanced and elaborated coping strategies in women with advancing age. These results are consistent with previous research that has shown an increase in psychological distress in people aged 24 and under [30]. Contrary to data from the general German population [44], living with a partner did not improve mental health outcomes in patients with endometriosis, presumably due to additional stress and burden in the relationship during the COVID-19 pandemic.

To assess the impact of the COVID-19 pandemic on the mental health of women with endometriosis, we examined several pandemic–related factors, such as the disruption of social networks, isolation or quarantine, and the duration of reduced social connections. There is a scarcity of data on the impact of isolation or quarantine duration on mental health outcomes. One previous study reported that social isolation lasting more than 10 days deteriorated mental health during the SARS-CoV-1 pandemic in Canada [31]. During the COVID-19 pandemic, however, duration of isolation or quarantine for more than 14 days proved to be an independent protective factor for the probability of a generalized anxiety disorder (GAD-2 ≥ 3), presumably due to a habituation effect. Nevertheless, during the current pandemic, the level of social network restriction was confirmed to be a significant independent risk factor for the probability of a generalized anxiety disorder (GAD ≥ 3) and overall mental symptom burden (PHQ-4 ≥ 6). These observations support previous findings that underlined the importance of social connection for well–being and life satisfaction [43] and identified social isolation and loneliness as risk factors for mental health issues [17,42,45]. Additionally, the government and the society should carefully balance the mental health risks imposed by social isolation with a relatively low benefit for the younger population, as the risk for excess death in high–income countries during the pandemic, such as Germany, was marginal [46].

We observed no evidence of diagnostic delay, time to onset of pain symptoms, or diagnosis having a significant independent effect on mental health outcomes. A similar study in Brazilian women with endometriosis also declared no link between the time from onset of pain symptoms or the time it took for the diagnosis of endometriosis for mental health [41]. In this study, higher age at diagnosis of endometriosis was independently correlated with lower symptom burden on the PHQ-4 scale (PHQ-4 ≥ 6), which may be related to less emotional suffering caused by the diagnosis of endometriosis at a later stage in life.

Chronic pain has been identified as a risk factor for adverse mental outcomes [47], and permanent pelvic pain has been recognized as a predictor of poor psychological health and increased perceived stress levels in women with and without endometriosis [11,38,48,49,50]. Based on our data, continuous pain, as well as the number of pain localization, were risk factors for several unfavorable psychological outcomes in univariate regression analyses. When other possible predictors, such as pain-induced disability, were considered, continuous pain and the number of pain localizations proved to be mediators of depression and anxiety but not independent predictors.

Physical disability has previously been linked to depressive disorders [42,51]. Pain-induced disability was recognized to be the most influential and reliable independent risk factor for mental health in our study population. The increased odds of self-reported anxiety and/or depression in women with higher levels of pain-related disability may be linked to the assumption of a higher risk of other endometriosis-associated comorbidities, such as infertility, or a more uncertain prognosis of the disease’s course and very personal development, such as sexual life and intimate relationships [38,52].

Our results also suggest that current (transient) fluctuations in pain intensity and pain-induced disability may have the potential to exert an immediate impact on mental health. Thus, proper management of physical complaints is essential to avoid adverse mental outcomes. Moreover, our findings confirm previous findings that indicated that the experience of pain and pain-induced disability rather than the mere diagnosis of endometriosis affected mental health [37,53]. Physical and mental conditions seem to influence one another [9]. In this analysis, we were unable to distinguish the causal direction of this effect, specifically whether increased levels of mental distress led to greater pain intensity or disability, or vice versa.

We identified resilience as an independent, potent, and reliable protective factor against the development of mental health symptoms in women with endometriosis. In our study group, high resilience significantly reduced the risk of anxiety, depression, and psychological burden. To date, the importance and influence of resilience in patients with endometriosis are poorly understood. Our findings support previous results, that resilience is a potent protective factor in the face of various adversities, such as chronic pain and stress [54,55,56].

### Limitations

We would like to discuss current findings in the context of some limitations. First, participants were recruited via an online survey, which may have resulted in a self-selection effect, as those with the highest level of pain-induced disability or mental distress might have responded to the survey. Nevertheless, a recent systematic review showed that samples obtained through Facebook were similarly representative of samples recruited via traditional methods [57]. Furthermore, because the patients answered the questionnaire directly, social desirability bias was greatly limited.

Second, we noticed a significant drop in the number of participants, with 413 accessing the questionnaire but only 274 responding to at least one of the mental health questions. The high drop-out rate may be related to a bias, as the patients who completed the questionnaire maybe those who were experiencing a greater degree of anxiety or depression at that time or who had a greater interest in the topic. Nevertheless, we assume that this fact did not affect the results of the study, as there were no significant differences in assessed clinical and demographic characteristics between those who did or did not respond to the questions concerning the assessed variables in the different predicting models for mental health.

Third, recall bias might have impacted the results of this study, as the evaluation of the symptoms preceding the pandemic may have been burdened by the psychological experience linked to the pandemic itself. However, recall is a common tool in the field of endometriosis, as diagnosis and treatment planning is guided substantially by retrospective pain assessment [58]. Previous findings showed that women with endometriosis were relatively accurate in their recall of pain [58].

Forth, as measured by the PHQ-4 questionnaire, self–reports were applied for the assessment of anxiety and depression. Moreover, the PHQ-4 is a screening tool rather than a diagnostic instrument for core depression and anxiety disorder symptoms in the previous two weeks [24,27]. There are few studies examining the concordance between clinical diagnosis and self-reported mental health measures. A study that evaluated the accuracy of self-reported instruments using structured interviews as reference standard showed that scales measuring mental health (BDI-II, HADS, and PHQ-9) had a sensitivity and a specificity of more than 69% and 72%, respectively, for predicting the clinical diagnosis [59]. As a result, self–reported levels of psychological distress may not always be validated by mental health professionals during a subsequent assessment. Moreover, women with endometriosis, who related only to information from the internet displayed higher levels of anxiety, as measured by a recent study using the Italian version of the STAI-Y6 questionnaire [60]. Nevertheless, as the women in our study were all diagnosed following a surgical procedure, we assume, that their information with respect to the disease does not relay only by internet research. Additionally, we might have used the STAI–Y6 questionnaire for assessing the trait anxiety, which was used in a current study of Italian women with endometriosis during the COVID-19 pandemic to assess the currently experienced anxiety [61]. Nevertheless, as the STAI-Y6 is not yet validated for the German language, we were not able to use this questionnaire.

Finally, the participants’ medication was not assessed, but previous studies have indicated a significant reduction in psychological symptoms, such as anxiety and depression, caused by pain reduction due to hormonal treatment [62,63]. Furthermore, significant reductions in depressive symptoms were described in chronic pain patients following the administration of pain relief medication [64]. Hence, the potential intake of hormonal treatments or analgesics by participants in this study had no negative impact on the psychological measures.

## 5. Conclusions

This study highlights the great vulnerability of chronic pain patients to mental health issues, as well as the intricate relationship between pain, pain-induced disability, and mental health. Our findings yield important clinical implications and support a multi-professional treatment regimen that addresses both physical and psychological complaints. The ability to identify women at risk for adverse psychological outcomes allows health care professionals to react quickly and establish necessary mental health support measures for each vulnerable individual. The ultra-brief PHQ-4 could be widely implemented even in busy outpatient care of general practitioners and gynecologists, as well as in inpatient care. Because the PHQ-4 is only a screening tool, the diagnosis must be confirmed using the appropriate DSM–V criteria. Furthermore, resilience was identified as a protective factor for depression and anxiety in women with endometriosis. Positive psychological interventions, such as resilience promoting strategies, may help to mitigate the negative impact of risk factors for adverse mental health outcomes in this population.

## Figures and Tables

**Table 1:** Differences between participants who did not complete the PHQ-4 questionnaire (group “Non-respondents”) versus those who completed at least one of the questions (group “Respondents”).

Variables	Values	Non-Respondents	Respondents	*p*-Value
**Demographic variables**				
Age	<25 y in % (n/N)	13.4% (16/119)	16.4% (45/274)	0.454 ^1^
	≥25 y in %(n/N)	86.8% (103/119)	83.6% (229/274)	
Having a stable relationship	No in % (n/N)	74.3% (78/105)	77.0% (211/274)	0.577 ^1^
	Yes in % (n/N)	25.7% (27/105)	23.0% (63/274)	
Living alone	No in % (n/N)	75.8% (91/120)	79.1% (216/273)	0.468 ^1^
	Yes in % (n/N)	24.2% (29/120)	20.9% (57/273)	
Educational level	Up to secondary level in % (n/N)	n.a.	29.4% (79/269)	n.a.
	Tertiary level in % (n/N)	n.a.	70.6% (190/269)	
**Pandemic-specific variables**				
Duration of i/q	<15 d in % (n/N)	16.3% (17/104)	10.2% (28/274)	0.100 ^1^
	≥15 d in % (n/N)	83.7% (87/104)	89.8% (246/274)	
Being in i/q	No in % (n/N)	5.9% (7/119)	2.6% (7/274)	0.102 ^1^
	Yes in % (n/N)	94.1% (112/119)	97.4% (267/274)	
Reduction of social network	No to moderate reduction in % (n/N)	31.4% (33/105)	27.4% (75/274)	0.434 ^1^
	Large reduction in % (n/N)	68.6% (72/105)	72.6% (199/274)	
**Endometriosis-specific variables**				
Time since diagnosis (y)	M (SD); N	3.95 (4.69); 98	4.42 (4.81); 273	0.184 ^2^
	Mdn (IQR)	2.00 (1.00–4.00)	3.00 (1.00–5.00)	
Age at diagnosis (y)	M (SD); N	28.16 (6.64); 98	27.64 (6.27); 273	0.347 ^2^
	Mdn (IQR)	28.00 (23.90–33.00)	27.00 (23.00–32.50)	
Time since pain onset (y)	M (SD); N	13.10 (7.51); 100	14.08 (7.90); 274	0.305 ^2^
	Mdn (IQR)	12.00 (7.00–18.00)	13.00 (8.00–20.00)	
Diagnostic delay (y)	M (SD); N	9.25 (6.79); 98	9.68 (6.94); 273	0.754 ^2^
	Mdn (IQR)	9.00 (4.00–13.90)	9.00 (4.50–14.00)	
Pain characteristics	Pain peaks in % (n/N)	63.6% (63/99)	65.3% (179/274)	0.762 ^1^
	Continuous pain in % (n/N)	36.4% (36/99)	34.7% (95/274)	
Number of pain localizations	M (SD); N	5.24 (0.88); 42	5.04 (1.19); 273	0.579 ^2^
	Mdn (IQR)	5.00 (5.00–6.00)	5.00 (5.00–6.00)	
**Pain intensity**				
Dysmenorrhoea prior to i/q	M (SD); N	62.80 (34.35); 40	65.41 (30.84); 244	0.829 ^2^
	Mdn (IQR)	72.50 (40.00–97.50)	73.50 (46.50–89.50)	
Non-cyclic pain prior to i/q	M (SD); N	45.52 (29.80); 42	51.99 (26.77); 261	0.134 ^2^
	Mdn (IQR)	41.00 (23.00–63.00)	51.00 (32.00–73.00)	
Dyspareunia prior to i/q	M (SD); N	43.19 (34.68); 37	44.64 (32.32); 247	0.815 ^2^
	Mdn (IQR)	31.00 (11.00–70.00)	45.00 (14.00–69.00)	
Dysuria prior i/q	M (SD); N	25.51 (31.28); 37	29.23 (29.06), 242	0.292 ^2^
	Mdn (IQR)	8.00 (2.00–47.00)	20.00 (4.00–48.00)	
Dyschezia prior to i/q	M (SD); N	37.26 (31.70); 39	41.02 (30.99); 254	0.449 ^2^
	Mdn (IQR)	29.00 (9.00–58.00)	37.00 (13.00–67.00)	
Lower back pain prior to i/q	M (SD); N	56.15 (31.48); 41	57.53 (32.57); 266	0.709 ^2^
	Mdn (IQR)	54.00 (33.00–89.00)	61.00 (33.00–88.00)	
Global pain prior to i/q	M (SD); N	43.81 (20.37); 33	47.70 (19.06); 204	0.253 ^2^
	Mdn (IQR)	45.50 (25.17–59.83)	47.83 (33.67–61.75)	
Current dysmenorrhoea	M (SD); N	34.11 (37.64); 9	60.75 (33.40); 248	**0.027 ^2^**
	Mdn (IQR)	12.00 (0.00–76.00)	70.50 (34.50–87.50)	
Current non-cyclic pain	M (SD); N	44.40 (33.08); 10	52.78 (30.22); 264	0.390 ^2^
	Mdn (IQR)	45.00 (8.00–61.00)	56.00 (27.00–78.00)	
Current dyspareunia	M (SD); N	48.00 (37.54); 9	43.89 (35.44); 248	0.618 ^2^
	Mdn (IQR)	57.00 (13.00–79.0)	44.00 (7.50–73.00)	
Current dysuria	M (SD); N	9.11 (14.29); 9	29.91 (31.19); 247	0.088 ^2^
	Mdn (IQR)	5.00 (1.00–8.00)	16.00 (2.00–16.00)	
Current dyschezia	M (SD); N	20.56 (23.16); 9	40.85 (32.46); 252	0.065 ^2^
	Mdn (IQR)	10.00 (2.00–36.00)	38.00 (10.00–67.50)	
Current lower back pain	M (SD); N	51.00 (38.08) 9	58.65 (33.98); 262	0.570 ^2^
	Mdn (IQR)	43.00 (24.00–76.00)	64.00 (28.00–88.00)	
Current global pain	M (SD); N	33.50 (22.06); 9	47.08 (21.18); 216	0.081 ^2^
	Mdn (IQR)	32.67 (14.67–54.00)	46.17 (31.33–62.50)	
**Pain-induced disability**				
Family prior to i/q	M (SD); N	5.56 (2.28); 40	5.13 (2.49); 274	0.263 ^2^
	Mdn (IQR)	5.50 (4.00–8.00)	5.00 (3.00–7.00)	
Recreational prior to i/q	M (SD); N	5.53 (2.55); 40	5.60 (2.56); 274	0.833 ^2^
	Mdn (IQR)	6.00 (4.00–7.50)	6.00 (4.00–8.00)	
Social activities prior to i/q	M (SD); N	5.62 (2.86); 40	5.45 (2.72); 274	0.690 ^2^
	Mdn (IQR)	6.00 (3.00–8.00)	6.00 (3.00–8.00)	
Occupational prior to i/q	M (SD); N	6.48 (2.58); 40	5.97 (2.81); 274	0.334 ^2^
	Mdn (IQR)	7.00 (4.00–8.00)	6.00 (4.00–8.00)	
Sexuality prior to i/q	M (SD); N	5.85 (3.21); 40	6.04 (3.27); 269	0.688 ^2^
	Mdn (IQR)	6.00 (3.00–8.50)	7.00 (3.00–9.00)	
Self-care prior to i/q	M (SD); N	2.95 (3.05); 40	2.71 (2.78); 274	0.801 ^2^
	Mdn (IQR)	2.50 (0.00–5.00)	2.00 (0.00–5.00)	
Life support prior to i/q	M (SD); N	2.73 (2.85); 40	2.67 (2.62); 274	0.864 ^2^
	Mdn (IQR)	2.00 (0.00–5.00)	2.00 (0.00–5.00)	
Discretional activities prior to i/q	M (SD); N	29.13 (10.49); 40	28.13 (11.22); 269	0.748 ^2^
	Mdn (IQR)	30.00 (23.00–37.50)	30.00 (21.00–37.00)	
Basic activities prior to i/q	M (SD)	5.67 (5.38); 40	5.38 (4.88); 269	0.995 ^2^
	Mdn (IQR)	5.00 (0.00–9.00)	4.00 (1.00–9.00)	
Global PDI prior to i/q	M (SD); N	34.80 (14.28); 40	33.51 (14.38); 269	0.817 ^2^
	Mdn (IQR)	33.50 (27.00–45.00)	34.00 (23.00–43.00)	
Current family activities	M (SD); N	4.13 (1.89); 8	5.34 (2.72); 273	0.166 ^2^
	Mdn (IQR)	4.50 (2.00–6.00)	5.00 (3.00–8.00)	
Current recreational activities	M (SD); N	3.38 (2.13); 8	5.37 (2.91); 273	**0.042 ^2^**
	Mdn (IQR)	4.00 (1.50–5.00)	6.00 (3.00–8.00)	
Current social activities	M (SD); N	2.75 (2.49); 8	4.56 (3.44); 272	0.112 ^2^
	Mdn (IQR)	3.50 (0.00–5.00)	5.00 (1.00–8.00)	
Current occupational activities	M (SD); N	3.00 (2.07); 8	5.36 (3.26); 272	**0.048 ^2^**
	Mdn (IQR)	4.00 (1.00–4.50)	5.00 (3.00–8.00)	
Current sexuality	M (SD); N	3.88 (2.90); 8	5.57 (3.51); 269	0.163 ^2^
	Mdn (IQR)	3.50 (2.50–4.50)	6.00 (3.00–9.00)	
Current self-care	M (SD); N	2.38 (2.33); 8	2.84 (2.86); 273	0.756 ^2^
	Mdn (IQR)	2.50 (0.00–4.00)	2.00 (0.00–5.00)	
Current life support	M (SD); N	2.25 (2.71); 8	2.67 (2.79); 273	0.596 ^2^
	Mdn (IQR)	1.50 (0.00–4.00)	2.00 (0.00–5.00)	
Current discretional activities	M (SD); N	17.13 (8.85); 8	26.31 (12.55); 269	**0.041 ^2^**
	Mdn (IQR)	20.00 (10.00–24.50)	27.00 (16.00–36.00)	
Current basic activities	M (SD); N	4.63 (4.98); 8	5.59 (5.13); 269	0.544 ^2^
	Mdn (IQR)	4.00 (0.00–8.00)	5.00 (1.00–9.00)	
Current global PDI	M (SD); N	21.75 (12.94); 10	31.90 (15.83); 269	0.083 ^2^
	Mdn (IQR)	23.50 (11.00–33.50)	32.00 (19.00–43.00)	
**Mental outcomes**				
PHQ-2	M (SD); N	n.a.	2.83 (1.689); 274	n.a.
	Mdn (IQR)	n.a.	2.00 (2.00–4.00)	
GAD-2	M (SD); N	n.a.	2.89 (1.827); 274	n.a.
	Mdn (IQR)	n.a.	2.00 (2.00–4.00)	
PHQ-4	M (SD); N	n.a.	5.72 (3.210); 274	n.a.
	Mdn (IQR)	n.a.	5.00 (3.00–8.00)	
BRS	M (SD); N	n.a.	2.75 (0.825); 273	n.a.
	Mdn (IQR)	n.a.	2.66 (2.16–3.33)	

BRS = brief resilience score; GAD-2 = Generalized Anxiety Disorder Scale; PHQ-2 = Patient Health Questionnaire for Depression; PHQ-4 = Patient Health Questionnaire for Depression and Anxiety; i/q = isolation or quarantine; d = days; N = Number of women for which data were available; n = sample size; M = mean; SD = standard deviation, Mdn = median; IQR: Interquartile Range; n.a. = not available/not applicable; y = years, Values in bold indicate statistical significance, as the level of statistical significance was set to *p* < 0.05 (^1^ = χ^2^-test; ^2^ = Mann-Whitney-U-test).

**Table 2:** Influence of demographic factors on self-reported mental health outcomes (univariate logistic regression analysis).

GAD-2 ≥ 3		GAD-2 ≥ 5		PHQ-2 ≥ 3		PHQ-2 ≥ 5		PHQ-4 ≥ 6		PHQ-4 ≥ 9	
*p*-Value	OR (95% CI)	*p*-Value	OR (95% CI)	*p*-Value	OR (95% CI)	*p*-Value	OR (95% CI)	*p*-Value	OR (95% CI)	*p*-Value	OR (95% CI)
**Age ≥ 25 years** (co: <25 years)											
0.449	0.781 (0.412–1.482)	0.374	0.723 (0.354–1.478)	0.053	0.526 (0.274–1.009)	0.192	0.619 (0.301–1.273)	0.053	0.527 (0.276–1.008)	0.339	0.705 (0.345–1.443)
**Having a partner** (co: not having a partner)											
0.698	0.894 (0.509–1.572)	0.322	0.701 (0.348–1.415)	0.901	0.965 (0.549–1.697)	0.843	0.933 (0.467–1.863)	0.554	0.842 (0.476–1.489)	0.808	0.920 (0.469–1.804)
**Living alone** (co: not living alone)											
0.668	1.136 (0.634–2.037)	0.881	1.053 (0.534–2.079)	0.063	1.751 (0.970–3.161)	0.908	0.959 (0.469–1.958)	0.177	1.498 (0.834–2.690)	0.565	1.217 (0.623–2.379)
**Tertiary educational level** (co: up to secondary educational level)											
**0.004**	**0.455** **(0.266–0.779)**	**0.025**	**0.510** **(0.283–0.918)**	**0.001**	**0.408** **(0.238–0.700)**	0.165	0.646 (0.348–1.197)	**<0.001**	**0.365** **(0.213–0.628)**	**0.003**	**0.411** **(0.228–0.741)**

GAD-2 = Generalized Anxiety Disorder Scale; PHQ-2 = Patient Health Questionnaire for Depression; PHQ-4 = Patient Health Questionnaire for Depression and Anxiety; OR = odds ratio; CI = confidence interval; co = controls; Values in bold indicate statistical significance, as the level of statistical significance was set to *p* < 0.05.

**Table 3:** Influence of pandemic-specific factors on self-reported mental health outcomes (univariate logistic regression analysis).

GAD-2 ≥ 3		GAD-2 ≥ 5		PHQ-2 ≥ 3		PHQ-2 ≥ 5		PHQ-4 ≥ 6		PHQ-4 ≥ 9	
*p*-Value	OR (95% CI)	*p*-Value	OR (95% CI)	*p*-Value	OR (95% CI)	*p*-Value	OR (95% CI)	*p*-Value	OR (95% CI)	*p*-Value	OR (95% CI)
**Duration of reduction of social network ≥ 15 days** (co: <15 days)											
0.165	0.568 (0.256–1.263)	0.525	0.754 (0.315–1.803)	0.713	0.864 (0.395–1.888)	0.638	0.804 (0.324–1.995)	0.831	0.918 (0.419–2.011)	0.493	0.737 (0.308–1.763)
**Being in i/q** (co: not being in i/q)											
0.632	0.691 (0.152–3.145)	0.559	1.892 (0.224–16.007)	0.999	0.000 (0.000)	0.640	1.665 (0.197–14.108)	0.501	0.594 (0.130–2.706)	0.742	0.756 (0.143–3.993)
**Large reduction of social network** (co: not at all to moderate reduction of social network)											
**0.014**	**1.986** **(1.149–3.433)**	0.375	1.342 (0.701–2.572)	0.172	1.455 (0.849–2.495)	0.961	1.016 (0.532–1.942)	**0.011**	**2.062** **(1.179–3.607)**	0.421	1.307 (0.681–2.506)

GAD-2 = Generalized Anxiety Disorder Scale; PHQ-2 = Patient Health Questionnaire for Depression; PHQ-4 = Patient Health Questionnaire for Depression and Anxiety; i/q = isolation or quarantine; OR = odds ratio; CI = confidence interval; co = controls; Values in bold indicate statistical significance, as the level of statistical significance was set to *p* < 0.05.

**Table 4:** Influence of endometriosis-specific variables on self-reported mental health outcomes (univariate logistic regression analysis).

GAD-2 ≥ 3		GAD-2 ≥ 5		PHQ-2 ≥ 3		PHQ-2 ≥ 5		PHQ-4 ≥ 6		PHQ-4 ≥ 9	
*p*-Value	OR (95% CI)	*p*-Value	OR (95% CI)	*p*-Value	OR (95% CI)	*p*-Value	OR (95% CI)	*p*-Value	OR (95% CI)	*p*-Value	OR (95% CI)
**Duration since diagnosis of endometriosis**											
0.818	1.006 (0.957–1.057)	0.263	0.963 (0.902–1.028)	0.933	0.998 (0.950–1.049)	0.668	0.986 (0.926–1.050)	0.793	1.007 (0.958–1.058)	0.568	0.982 (0.924–1.045)
**Age at diagnosis of endometriosis**											
0.341	0.982 (0.945–1.020)	0.944	1.002 (0.958–1.047)	0.130	0.971 (0.934–0.009)	0.362	0.979 (0.934–1.025)	**0.047**	**0.961** **(0.924–0.999)**	0.959	1.001 (0.957–1.047)
**Duration since pain onset**											
0.734	0.995 (0.965–1.025)	0.540	1.011 (0.976–1.047)	0.943	1.001 (0.971–1.032)	0.582	0.990 (0.953–1.027)	0.645	0.993 (0.963–1.023)	0.436	1.014 (0.979–1.050)
**Diagnostic delay**											
0.630	0.992 (0.958–1.026)	0.150	1.030 (0.990–1.071)	0.836	1.004 (0.970–1.039)	0.831	0.995 (0.954–1.038)	0.524	0.989 (0.955–1.024)	0.212	1.026 (0.986–1.068)
**Pain characteristics: continuous pain** (co: pain peaks)											
0.571	1.155 (0.702–1.900)	**0.027**	**1.899** **(1.075–3.354)**	**0.003**	**2.134** **(1.286–3.540)**	**0.009**	**2.182** **(1.213–3.925)**	0.146	1.449 (0.879–2.390)	**0.004**	**2.333** **(1.317–4.134)**
**Number of pain localizations**											
0.073	1.208 (0.983–1.485)	0.312	1.138 (0.885–1.464)	**<0.001**	**1.509** **(1.202–1.895)**	**0.017**	**1.442** **(1.067–1.949)**	**0.019**	**1.293** **(1.043–1.604)**	0.086	1.264 (0.968–1.651)

GAD-2 = Generalized Anxiety Disorder Scale; PHQ-2 = Patient Health Questionnaire for Depression; PHQ-4 = Patient Health Questionnaire for Depression and Anxiety; OR = odds ratio; CI = confidence interval; co = controls; Values in bold indicate statistical significance, as the level of statistical significance was set to *p* < 0.05.

**Table 5:** Influence of previous and current pain intensity on self-reported mental health outcomes (univariate logistic regression analysis).

	GAD-2 ≥ 3		GAD-2 ≥ 5		PHQ-2 ≥ 3		PHQ-2 ≥ 5		PHQ-4 ≥ 6		PHQ-4 ≥ 9	
	*p*-Value	OR (95% CI)	*p*-Value	OR (95% CI)	*p*-Value	OR (95% CI)	*p*-Value	OR (95% CI)	*p*-Value	OR (95% CI)	*p*-Value	OR (95% CI)
**Dysmenorrhea**												
VAS_P_	0.063	1.008 (1.000–1.016)	0.303	1.005 (0.995–1.015)	**0.008**	**1.012** **(1.003–1.021)**	0.066	1.010 (0.999–1.021)	**0.013**	**1.011** **(1.002–1.020)**	0.063	1.010 (0.999–1.021)
VAS_C_	**<0.001**	**1.015** **(1.007–1.023)**	**0.009**	**1.013** **(1.003–1.023)**	**<0.001**	**1.019** **(1.010–1.027)**	**0.004**	**1.016** **(1.005–1.026)**	**<0.001**	**1.018** **(1.010–1.026)**	**<0.001**	**1.020** **(1.009–1.031)**
**Non-cyclic pain**												
VAS_P_	0.749	1.001 (0.992–1.011)	0.537	1.003 (0.993–1.014)	**0.002**	**1.015** **(1.006–1.025)**	0.063	1.011 (0.999–1.022)	0.063	1.009 (1.000–1.018)	0.137	1.008 (0.997–1.019)
VAS_C_	**0.004**	**1.012** **(1.004–1.021)**	**0.025**	**1.011** **(1.001–1.021)**	**<0.001**	**1.023** **(1.014–1.032)**	**0.001**	**1.019** **(1.008–1.030)**	**<0.001**	**1.016** **(1.008–1.025)**	**<0.001**	**1.021** **(1.010–1.031)**
**Dyspareunia**												
VAS_P_	0.586	1.002 (0.994–1.010)	0.071	1.008 (0.999–1.018)	**0.015**	**1.010** **(1.002–1.018)**	0.176	1.006 (0.997–1.016)	0.145	1.006 (0.998–1.014)	0.052	1.009 (1.000–1.018)
VAS_C_	0.173	1.005 (0.998–1.012)	0.061	1.008 (1.000–1.016)	**0.001**	**1.013** **(1.005–1.020)**	**0.049**	**1.009** **(1.000–1.017)**	**0.033**	**1.008** **(1.001–1.015)**	**0.011**	**1.011** **(1.003–1.020)**
**Dysuria**												
VAS_P_	0.827	1.001 (0.992–1.010)	0.263	1.006 (0.996–1.016)	**0.003**	**1.014** **(1.005–1.023)**	0.369	1.005 (0.995–1.015)	0.190	1.006 (0.997–1.015)	0.231	1.006 (0.996–1.016)
VAS_C_	0.945	1.000 (0.992–1.008)	0.668	1.002 (0.993–1.011)	**0.003**	**1.012** **(1.004–1.021)**	0.237	1.006 (0.996–1.015)	0.238	1.005 (0.997–1.013)	0.179	1.006 (0.997–1.015)
**Dyschezia**												
VAS_P_	0.895	0.999 (0.992–1.007)	0.916	0.999 (0.990–1.009)	**0.047**	**1.008** **(1.000–1.016)**	0.271	1.005 (0.996–1.015)	0.518	1.003 (0.995–1.011)	0.283	1.005 (0.996–1.014)
VAS_C_	**0.004**	**1.012** **(1.004–1.020)**	0.409	1.004 (0.995–1.013)	**<0.001**	**1.019** **(1.011–1.028)**	**0.009**	**1.012** **(1.003–1.022)**	**<0.001**	**1.015** **(1.007–1.023)**	**0.011**	**1.012** **(1.003–1.021)**
**Lower back pain**												
VAS_P_	0.164	1.005 (0.998–1.013)	0.073	1.008 (0.999–1.017)	**0.001**	**1.013** **(1.005–1.020)**	0.269	1.005 (0.996–1.014)	**0.005**	**1.011** **(1.003–1.019)**	0.132	1.007 (0.998–1.016)
VAS_C_	**0.036**	**1.008** **(1.000–1.015)**	0.149	1.006 (0.998–1.015)	**<0.001**	**1.016** **(1.008–1.024)**	0.287	1.005 (0.996–1.014)	**0.004**	**1.011** **(1.004–1.019)**	**0.024**	**1.010** **(1.001–1.019)**
**Global pain experience**												
VAS_P_	0.380	1.007 (0.992–1.021)	0.102	1.014 (0.997–1.032)	**<0.001**	**1.036** **(1.019–1.053)**	**0.025**	**1.020** **(1.003–1.038)**	**0.008**	**1.021** **(1.005–1.036)**	**0.018**	**1.021** **(1.004–1.039)**
VAS_C_	**0.002**	**1.021** **(1.008–1.035)**	**0.017**	**1.019** **(1.003–1.034)**	**<0.001**	**1.047** **(1.030–1.063)**	**0.001**	**1.028** **(1.011–1.044)**	**<0.001**	**1.031** **(1.016–1.045)**	**<0.001**	**1.034** **(1.017–1.051)**

GAD-2 = Generalized Anxiety Disorder Scale; PHQ-2 = Patient Health Questionnaire for Depression; PHQ-4 = Patient Health Questionnaire for Depression and Anxiety; i/q = isolation or quarantine; OR = odds ratio; CI = confidence interval; VAS_P_ = previous pain level, continuous variable; VAS_C_ = current pain level, continuous variable; co = controls; Values in bold indicate statistical significance, as the level of statistical significance was set to *p* < 0.05.

**Table 6:** Influence of pain-induced disability on self–reported mental health outcomes (univariate logistic regression analysis).

	GAD-2 ≥ 3		GAD-2 ≥ 5		PHQ-2 ≥ 3		PHQ-2 ≥ 5		PHQ-4 ≥ 6		PHQ-4 ≥ 9	
	*p*-Value	OR (95% CI)	*p*-Value	OR (95% CI)	*p*-Value	OR (95% CI)	*p*-Value	OR (95% CI)	*p*-Value	OR (95% CI)	*p*-Value	OR (95% CI)
**Family**												
Previous i/q	0.251	1.058 (0.961–1.164)	**0.039**	**1.131** **(1.006–1.271)**	**<0.001**	**1.220** **(1.102–1.351)**	0.051	1.128 (0.999–1.273)	**0.005**	**1.153** **(1.044–1.273)**	**0.016**	**1.158** **(1.028–1.305)**
Current	**<0.001**	**1.204** **(1.097–1.321)**	**0.001**	**1.221** **(1.091–1.367)**	**<0.001**	**1.346** **(1.215–1.490)**	**<0.001**	**1.326** **(1.170–1.502)**	**<0.001**	**1.301** **(1.178–1.438)**	**<0.001**	**1.374** **(1.212–1.558)**
**Recreational**												
Previous i/q	0.706	1.018 (0.928–1.117)	0.086	1.105 (0.986–1.237)	**0.011**	**1.133** **(1.029–1.247**)	0.158	1.088 (0.968–1.222)	0.098	1.083 (0.985–1.191)	**0.048**	**1.123** **(1.001–1.261)**
Current	**<0.001**	**1.191** **(1.091–1.299)**	**0.002**	**1.181** **(1.064–1.311)**	**<0.001**	**1.292** **(1.177–1.420)**	**<0.001**	**1.267** **(1.129–1.422)**	**<0.001**	**1.235** **(1.128–1.353)**	**<0.001**	**1.284** **(1.146–1.439)**
**Social activities**												
Previous i/q	0.202	1.059 (0.970–1.156)	**0.007**	**1.165** **(1.044–1.300)**	**<0.001**	**1.202** **(1.095–1.320)**	0.125	1.089 (0.977–1.215)	**0.006**	**1.136** **(1.037–1.244)**	**0.009**	**1.157** **(1.037–1.292)**
Current	**<0.001**	**1.128** **(1.050–1.211)**	**0.001**	**1.151** **(1.057–1.254**)	**<0.001**	**1.241** **(1.149–1.341)**	**<0.001**	**1.213** **(1.106–1.331)**	**<0.001**	**1.193** **(1.107–1.285)**	**<0.001**	**1.229** **(1.122–1.347)**
**Occupational**												
Previous i/q	0.058	1.087 (0.997–1.184)	**0.035**	**1.119** **(1.008–1.242)**	**<0.001**	**1.237** **(1.127–1.358)**	**0.043**	**1.118** **(1.003–1.246)**	**<0.001**	**1.189** **(1.086–1.302)**	**0.004**	**1.172** **(1.051–1.306)**
Current	**<0.001**	**1.193** (**1.103–1.291)**	**0.005**	**1.139** **(1.041–1.246)**	**<0.001**	**1.305** **(1.198–1.421)**	**<0.001**	**1.226** **(1.110–1.354)**	**<0.001**	**1.273** **(1.171–1.384)**	**<0.001**	**1.268** **(1.148–1.402)**
**Sexuality**												
Previous i/q	0.817	1.009 (0.937–1.085)	**0.021**	**1.115** **(1.016–1.223**)	**0.022**	**1.091** **(1.012–1.177)**	**0.035**	**1.109** **(1.007–1.221)**	0.102	1.064 (0.988–1.147)	**0.031**	**1.108** **(1.010–1.216)**
Current	0.058	1.069 (0.998–1.146)	**0.005**	**1.131** **(1.038–1.232)**	**<0.001**	**1.155** **(1.074–1.241)**	**0.001**	**1.175** **(1.070–1.290)**	**0.002**	**1.120** **(1.043–1.203)**	**0.001**	**1.158** **(1.059–1.266)**
**Self-care**												
Previous i/q	0.215	1.056 (0.969–1.150)	0.186	1.068 (0.969–1.178)	**<0.001**	**1.227** **(1.118–1.346)**	**0.006**	**1.152** **(1.042–1.273)**	**0.005**	**1.136** **(1.040–1.240)**	**0.010**	**1.136** **(1.031–1.253)**
Current	**0.024**	**1.103** **(1.013–1.201)**	0.073	1.090 (0.992–1.198)	**<0.001**	**1.297** **(1.179–1.428)**	**<0.001**	**1.194** **(1.083–1.317)**	**<0.001**	**1.198** **(1.096–1.309)**	**0.001**	**1.170** **(1.064–1.286)**
**Life support**												
Previous i/q	0.616	1.023 (0.935–1.121)	0.368	1.049 (0.945–1.165)	**0.011**	**1.128** **(1.028–1.238)**	0.445	1.043 (0.936–1.162)	0.110	1.077 (0.983–1.181)	0.206	1.070 (0.964–1.187)
Current	0.075	1.082 (0.992–1.179)	0.469	1.037 (0.940–1.144)	**<0.001**	**1.196** **(1.092–1.310)**	0.090	1.090 (0.987–1.204)	**0.009**	**1.124** **(1.030–1.227)**	0.054	1.100 (0.999–1.212)
**Discretional activities**												
Previous i/q	0.166	1.015 (0.994–1.038)	**0.005**	**1.040** **(1.012–1.069)**	**<0.001**	**1.054** **(1.029–1.079)**	**0.015**	**1.036** **(1.007–1.066)**	**0.001**	**1.039** **(1.015–1.063)**	**0.002**	**1.047** **(1.017–1.077)**
Current	**<0.001**	**1.047** **(1.026–1.069)**	**<0.001**	**1.049** **(1.023–1.075)**	**<0.001**	**1.081** **(1.056–1.107)**	**<0.001**	**1.077** **(1.047–1.109)**	**<0.001**	**1.067** **(1.043–1.091)**	**<0.001**	**1.083** **(1.052–1.115)**
**Basic activities**												
Previous i/q	0.253	1.029 (0.980–1.081)	0.180	1.039 (0.982–1.100)	**<0.001**	**1.114** **(1.057–1.174)**	**0.026**	**1.068** **(1.008–1.132)**	**0.008**	**1.071** **(1.018–1.126)**	**0.018**	**1.071** **(1.012–1.133)**
Current	**0.031**	**1.054** **(1.005–1.105)**	0.204	1.035 (0.981–1.092)	**<0.001**	**1.144** **(1.085–1.207)**	**0.003**	**1.087** **(1.029–1.148)**	**<0.001**	**1.094** **(1.042–1.150)**	**0.004**	**1.082** **(1.026–1.142)**
**Global PDI**												
Previous i/q	0.142	1.013 (0.996–1.030)	**0.009**	**1.028** **(1.007–1.050**)	**<0.001**	**1.046** **(1.027–1.066)**	**0.008**	**1.030** **(1.008–1.052)**	**0.001**	**1.032** **(1.013–1.050)**	**0.001**	**1.036** **(1.014–1.059)**
Current	**<0.001**	**1.035** **(1.018–1.052)**	**0.001**	**1.033** **(1.014–1.052)**	**<0.001**	**1.067** **(1.047–1.088)**	**<0.001**	**1.055** **(1.033–1.078)**	**<0.001**	**1.052** **(1.034–1.071)**	**<0.001**	**1.058** **(1.036–1.080)**

GAD-2 = Generalized Anxiety Disorder Scale; PHQ-2 = Patient Health Questionnaire for Depression; PHQ-4 = Patient Health Questionnaire for Depression and Anxiety; i/q = isolation or quarantine; OR = odds ratio; CI = confidence interval; PDI_C_ = current disability, continuous variable; Values in bold indicate statistical significance, as the level of statistical significance was set to *p* < 0.05.

**Table 7:** Influence of resilience on self-reported mental health outcomes (univariate logistic regression analysis).

	GAD-2 ≥ 3		GAD-2 ≥ 5		PHQ-2 ≥ 3		PHQ-2 ≥ 5		PHQ-4 ≥ 6		PHQ-4 ≥ 9	
	*p*-Value	OR (95% CI)	*p*-Value	OR (95% CI)	*p*-Value	OR (95% CI)	*p*-Value	OR (95% CI)	*p*-Value	OR (95% CI)	*p*-Value	OR (95% CI)
**BRS (cv)**	**<0.001**	**0.253** **(0.170–0.376)**	**<0.001**	**0.214** **(0.132–0.349)**	**<0.001**	**0.424** **(0.303–0.593)**	**<0.001**	**0.303** **(0.194–0.475)**	**<0.001**	**0.278** **(0.189–0.410)**	**<0.001**	**0.247** **(0.155–0.395)**
**BRS > 2.66**	**<0.001**	**0.240** **(0.145–0.399)**	**<0.001**	**0.157** **(0.077–0.317)**	**<0.001**	**0.361** **(0.220–0.592)**	**<0.001**	**0.302** **(0.158–0.575)**	**<0.001**	**0.221** **(0.132–0.371)**	**<0.001**	**0.231** **(0.120–0.443)**
**BRS > 3.33**	**<0.001**	**0.110** **(0.05–0.234)**	**<0.001**	**0.073** **(0.017–0.306)**	**<0.001**	**0.312** **(0.169–0.579)**	**0.003**	**0.236** **(0.090–0.619)**	**<0.001**	**0.157** **(0.076–0.325)**	**<0.001**	**0.116** **(0.035–0.383)**

OR = odds ratio; CI = confidence interval, BRS = Brief Resilience Score; GAD-2 = Generalized Anxiety Disorder Scale; PHQ-2 = Patient Health Questionnaire for Depression; PHQ-4 = Patient Health Questionnaire for Depression and Anxiety; co = controls; cv = continuous variable; Values in bold indicate statistical significance, as the level of statistical significance was set to *p* < 0.05.

## Data Availability

The datasets analyzed during the current study are available from the corresponding author on reasonable request.

## Supplementary Materials

The following supporting information can be downloaded at: https://www.mdpi.com/article/10.3390/ijerph19084927/s1, Table S1: Mental scores by sample characteristics of previous publications.
