# Supplementary material for: Predictors of Psychological Distress in Women with Endometriosis during the COVID-19 Pandemic
Source: Int J Environ Res Public Health. 2022 Apr 18;19(8):4927. doi: 10.3390/ijerph19084927 (PMC9024970; doi:10.3390/ijerph19084927)
Supplement: Supplementary file 1 [file ijerph-19-04927-s001.zip › ijerph-1657086-supplementary.pdf]

Supplementary Table S1: Mental scores by sample characteristics of previous publications.

Participant sample	Values	Representative survey of the German population [20]	Representative survey of the German population [34]				German patients with fibromyalgia [35]
			No chronic pain	Chronic localized pain	Chronic oligo-localized pain	Chronic multi-localized pain	
PHQ-2	M (SD)	0.94 (1.20)	0.47 (0.82)	0.79 (0.96)	1.08 (1.13)	1.97 (1.49)	3.0 (1.7)
	N	5010	1684	222	458	146	1620
GAD-2	M (SD)	0.82 (1.10)	0.29 (0.69)	0.51 (0.87)	0.73 (1.05)	1.38 (1.42)	2.9 (1.8)
	N	5027	1684	222	458	146	1617
PHQ-4	M (SD)	1.76 (2.06)	n.a.	n.a.	n.a.	n.a.	5.9 (3.2)
	N	5003	n.a.	n.a.	n.a.	n.a.	1617

GAD-2 = Generalized Anxiety Disorder Scale; PHQ-2 = Patient Health Questionnaire for Depression; PHQ-4 = Patient Health Questionnaire for Depression and Anxiety; M = Mean; N = Number of women for which data were available; SD = standard deviation; n.a. = not available.
